# Theoretical Prediction of Structures, Vibrational Circular Dichroism, and Infrared Spectra of Chiral Be_4_B_8_ Cluster at Different Temperatures

**DOI:** 10.3390/molecules26133953

**Published:** 2021-06-28

**Authors:** Carlos Emiliano Buelna-García, Eduardo Robles-Chaparro, Tristan Parra-Arellano, Jesus Manuel Quiroz-Castillo, Teresa del-Castillo-Castro, Gerardo Martínez-Guajardo, Cesar Castillo-Quevedo, Aned de-León-Flores, Gilberto Anzueto-Sánchez, Martha Fabiola Martin-del-Campo-Solis, Ana Maria Mendoza-Wilson, Alejandro Vásquez-Espinal, Jose Luis Cabellos

**Affiliations:** 1 Departamento de Investigación en Polímeros y Materiales, Edificio 3G, Universidad de Sonora, Hermosillo 83000, Sonora, Mexico; a209205768@unison.mx (C.E.B.-G.); jesus.quiroz@unison.mx (J.M.Q.-C.); teresa.delcastillo@unison.mx (T.d.-C.-C.); 2 Organización Científica y Tecnológica del Desierto, Hermosillo 83150, Sonora, Mexico; 3 Departamento de Ciencias Químico Biologicas, Edificio 5A, Universidad de Sonora, Hermosillo 83000, Sonora, Mexico; a214201767@unison.mx (E.R.-C.); a215207594@unison.mx (T.P.-A.); aned.deleon@unison.mx (A.d.-L.-F.); 4 Unidad Académica de Ciencias Químicas, Área de Ciencias de la Salud, Universidad Autónomade Zacatecas, Km. 6 Carretera Zacatecas-Guadalajara s/n, Ejido La Escondida C.P., Zacatecas 98160, Zac, Mexico; germtzguajardo@uaz.edu.mx; 5 Departamento de Fundamentos del Conocimiento, Centro Universitario del Norte, Universidad de Guadalajara, Carretera Federal No. 23, Km. 191, C.P., Colotlán 46200, Jalisco, Mexico; castillo.quevedo@cunorte.udg.mx (C.C.-Q.); mfmartindelcampo@cunorte.udg.mx (M.F.M.-d.-C.-S.); 6 Centro de Investigaciones en Óptica, A.C., León 37150, Guanajuato, Mexico; gilberto.anzueto@cio.mx; 7 Coordinación de Tecnología de Alimentos de Origen Vegetal, CIAD, A.C., Carretera Gustavo Enrique Astiazarán Rosas, No. 46, Hermosillo 83304, Sonora, Mexico; mwilson@ciad.mx; 8 Computational and Theoretical Chemistry Group Departamento de Ciencias Químicas, Facultad de Ciencias Exactas, Universidad Andres Bello, Republica 498, Santiago 8370035, Chile; a.vasquezespinal@uandresbello.edu; 9 Departamento de Investigación en Física, Edificio 3M, Universidad de Sonora, Hermosillo 83000, Sonora, Mexico

**Keywords:** global minimum, beryllium–boron cluster, Be_4_B_8_, density functional theory, temperature, Boltzmann factors, Gibbs free energy, entropy, enthalpy, thermochemistry, vibrational circular dichroism, IR spectra, quantum statistical mechanics, genetic algorithm, adaptive natural density partitioning method, nanothermodynamics

## Abstract

Lowest-energy structures, the distribution of isomers, and their molecular properties depend significantly on geometry and temperature. Total energy computations using DFT methodology are typically carried out at a temperature of zero K; thereby, entropic contributions to the total energy are neglected, even though functional materials work at finite temperatures. In the present study, the probability of the occurrence of one particular Be_4_B_8_ isomer at temperature T is estimated by employing Gibbs free energy computed within the framework of quantum statistical mechanics and nanothermodynamics. To identify a list of all possible low-energy chiral and achiral structures, an exhaustive and efficient exploration of the potential/free energy surfaces is carried out using a multi-level multistep global genetic algorithm search coupled with DFT. In addition, we discuss the energetic ordering of structures computed at the DFT level against single-point energy calculations at the CCSD(T) level of theory. The total VCD/IR spectra as a function of temperature are computed using each isomer’s probability of occurrence in a Boltzmann-weighted superposition of each isomer’s spectrum. Additionally, we present chemical bonding analysis using the adaptive natural density partitioning method in the chiral putative global minimum. The transition state structures and the enantiomer–enantiomer and enantiomer–achiral activation energies as a function of temperature evidence that a change from an endergonic to an exergonic type of reaction occurs at a temperature of 739 K.

## 1. Introduction

The potential of boron atoms to form stable molecular networks [1,2] lies in the fact that they have three valence electrons and four available orbitals, which implies they are electron-deficient. In addition, they have a small covalent radius of 0.8–1.01 Å [3,4], a high ionization energy (344.2 kJ/mol) [3], and an affinity for oxygen atoms, which is the basis of borates [3,5]. Boron atoms’ electron deficiency gives rise to a vast number of allotropic forms and uncommon geometries [2,6,7], such as nanotubes [8,9], borospherenes [10], borophene [7], cages [9,11], planar [12], quasi planar [13], rings [14,15], chiral [13,16,17,18,19,20], boron-based helix clusters [16,21], and fluxional boron clusters [2,21,22,23,24,25,26,27,28,29,30,31], which have recently attracted the interest of experimental and theoretical researchers.

Since molecular properties depend greatly on their geometry [32,33], boron clusters exhibit a large number of molecular properties that have potential applications in medicine [34,35,36,37], molecular motors [21,23,38], superhard materials [39], hydrogen storage [40], batteries [41,42,43,44], catalysis [45], and energy materials [46], among many others.

In particular, these nanoclusters have attracted attention due to their chiroptical properties, potential applications in efficient chiral discrimination [47,48], nonlinear optics [49], and potential to create chiral materials with interesting properties [13,50,51], not to mention the fact that chiral structures play a decisive role in biological activity [52]. Previous theoretical studies, together with experimental photoelectron spectroscopy, have reported the first pure boron chiral B_30_^−^ structure as the putative global minimum [13]. In these pairs of planar enantiomers, chirality arises due to the hexagonal hole and its position. A year later, the lowest-energy structures of the B_39_^−^ borospherene were reported as chiral due to their hexagonal and pentagonal holes [17]. Similarly, the B_44_ cluster was reported as a chiral structure due to its nonagonal holes [20]. In these clusters, holes in the structure cause chirality.

Beryllium-doped boron clusters exhibit remarkable properties such as fluxionality [7,21,31,53,54,55] and aromaticity [21,56], as well as characteristics similar to borophene [57]. Previous theoretical studies have shown that the boron fullerenes B_60_ and B_80_ can be stabilized by surrounding the boron clusters with beryllium atoms [58,59], which effectively compensates for boron electronic deficiency [59]. These effects make beryllium-doped boron clusters interesting research objects. Particularly attractive are Be_6_B_11_^−^ chiral helices, as reported by Gou et al. [21], Yanez et al. [18], and Garcia-Buelna et al. [33], as a low-lying and fluxional isomer. Later, chemical bonding and the mechanism of formation studies of the beryllium-doped boron chiral cluster Be_6_B_10_^2−^ and coaxial triple-layered Be_6_B_10_^2−^ sandwich structure were reported [16,55]. In these structures, chirality arises due to the formation of a boron helix. However, only a few theoretical studies have been carried out on vibrational circular dichroism (VCD) and infrared spectroscopy (IR) as a function of temperature in beryllium–boron clusters [33,60]. We emphasize that there are neither theoretical nor experimental studies of VCD/IR spectra in chiral Be_4_B_8_ clusters, although VCD/IR spectra give insight into the geometrical structure [61,62,63,64]. Recently, Castiglioni et al. reviewed experimental aspects of solid-state circular dichroism [65], and Avilés-Moreno et al. reported the experimental and theoretical IR/VCD spectra of various compounds [66,67,68,69]. VCD is differential spectroscopy sensitive to the difference in the absorption for the left and right polarized light [61,64,70]. It usually is four times in magnitude smaller than IR absorption [66] and yields information on the lowest energy conformation in solution [71,72]; thus, the chiral molecule’s absolute configuration can be determined employing the VCD spectra. [63,73,74,75].

IR frequencies are related to the second derivative of the potential energy and are useful in identifying transition states and computing thermodynamics through the vibrational partition function [33,76,77]. The structure of neutral boron clusters B_11_, B_16_, and B_17_ was probed by IR [78].

The DFT VCD/IR spectra depend on the functional and basis set employed [63] and significantly on the lowest-energy achiral and chiral structures, so we need to efficiently sample the free energy surface to find the distribution of isomers at different temperatures [32,33,79,80,81]. A considerable change in the isomer distribution and the energetic separation among them are the first notable effects of temperature [33]. Useful materials work at finite temperatures; in those conditions, Gibbs free energy is minimized [82] and determines the putative global minimum at a given temperature [33], whereas the entropy of the atomic cluster is maximized [82].

Although Mermin et al. [83] studied the thermal properties of inhomogeneous electron gas in the mid-1960s, most DFT calculations are typically performed at a temperature of zero. Recently, DFT was extended to a finite temperature [84,85,86], but as far as we know, it has not been implemented in any software; however, molecular dynamics and other simulation tools have been employed to study atomic clusters at finite temperatures [27,29,87,88,89,90].

In this study, based on the Gibbs free energy of each isomer, we computed the probability of occurrence of each particular isomer of Be_4_B_8_ as a function of temperature using quantum statistical mechanics. The computed VCD/IR spectrum of each isomer is multiplied by their corresponding Boltzmann weight at temperature T; then, they are summed together to produce a final Boltzmann-weighted spectrum. In the mid-1980, P. J. Stephens, with co-workers, implemented the atomic axial tensors in Gaussian 80 code to compute the VCD spectrum of propylene oxide and compare it with the experimental spectrum [91]. Later, Nafie and Stephens employed the Boltzmann weights scheme. They computed the VCD spectrum for each isomer, and the total resulting spectra were averaged and weighted by Boltzmann factors [61,92,93,94]. Recently, these factors were used in other previous works [33,79,80,81,84,95].

To achieve the mentioned above, we located all low-energy structures on the potential and free energy surfaces of the Be_4_B_8_ cluster with a genetic algorithm coupled to DFT and computed the Boltzmann weights at temperatures ranging from 20 to 1900 K. We also located the solid–solid transformation point at 739 K, where chiral and achiral structures coexist, and computed the energy barrier (Ea) for temperatures ranging from 20 to 1900 K for the transformation of enantiomers (plus, P) to an achiral structure. In addition, the energy of enantiomerization was computed between P and minus (M) enantiomers. We investigated how the symmetry point group affects the Gibbs free energy. Our results show that the chirality on Be_4_B_8_ arises from Be–Be dimers capping the boron ring; thus, the lowest energy chiral structure is favored by the interaction between beryllium and the boron framework. The high energy of the enantiomerization of the Be_4_B_8_ cluster at temperatures ranging from 20 to 1900 K suggests that it is a good candidate for use in various applications in medicine; in only one of the enantiomers showed the desired effect. The computed formation enthalpy (AH) between the chiral and achiral structure at 739 K shows that there is a change from an endothermic to exothermic reaction. Our results indicate that the Boltzmann-weighted VCD spectrum is zero at all ranges of temperatures, whereas the Boltzmann IR-weighted spectrum is strongly dominated by the lowest-energy pair of enantiomers.

The remainder of the manuscript is organized as follows: Section 2 gives the computational details and a brief overview of the theory and algorithms used. The results and discussion are presented in Section 3. We discuss the effect of the symmetry in the energetic ordering and clarify the origin of the 0.41 kcal/mol difference in energy between two structures with symmetries C_2_ and C_1_ that appear when we compute the Gibbs free energy. A comparison among the energies computed at a single point CCSD(T) against the DFT levels of theory and the T_1_ diagnostic is presented. The interconversion energy barriers between P and M enantiomers and between an achiral structure and P-enantiomer are discussed in terms of temperature. IR spectra are analyzed as a function of temperature. Conclusions are given in Section 4.

## 2. Theoretical Methods and Computational Details

### 2.1. Global Minimum Search and Computational Details

For theoretical studies of an atomic cluster, the first step is locating the putative global minimum and all the closest low-energy structures on its potential/free energy surface, since the measured molecular properties are statistical averages over a range of conformations [33,96]. We must keep in mind that experimental atomic molecular studies are conducted in non-zero temperatures, while theoretical studies based on density functional theory computations are typically performed at 0 K [33,97]. So, a systematic and exhaustive exploration of the multidimensional potential/free energy surface is needed to avoid the incomplete sampling of the configuration space [33,79,80]. It is important to consider all low-energy structures as relative populations to account for the molecular properties with a weighted Boltzmann factors [33,79,80].

Several algorithms to explore the potential/free energy surface coupled to an any electronic structure package have been successfully employed so far, such as AIRSS approach, [98] simulated annealing, [94,95,96,97,98,99,100,101,102,103,104], kick methodology [105,106,107,108,109,110,111,112,113,114,115,116], and genetic algorithms [9,21,33,112,116,117,118,119], among others.

Our computational procedure to explore the potential/free energy surface of the Be_4_B_8_ cluster employs a genetic algorithm implemented in GALGOSON code [33]. This methodology consists of a multi-step approach (cascade) to efficiently sample the potential/free energy surface coupled to the Gaussian 09 code [120].

Our multi-step strategy employs more accurate levels of theory applied to each step to arrive at the most stable lowest-lying isomers. In the first step of our methodology, the code builds an initial random population of 3D structures (two hundred structures per atom of the Be_4_B_8_ cluster), employing a strategy used in previous works [21,33,113,116,117,118,121,122,123]. The first optimization was carried out at the PBE0 [124]/LANL2DZ [125] level. The algorithm stops if the lowest-energy structure persists for five generations. All isomers lying below 20 kcal/mol are re-optimized at the PBE0 [124]/def2TZVP [126,127] level, including Grimme’s dispersion effects (GD3) [128], as implemented in Gaussian 09 code. In total, at this point and in all previous stages, about 2800 relaxations to a local-energy minimum are performed.

Additionally, we make sure that the lowest vibrational mode of each isomer is positive in order to identify a valid energy minimum. In the final step, single-point (SP) energy calculations for the low-energy structures lying below 10 kcal/mol are carried out at the CCSD(T)/def2-TZVP//PBE0-D3/def2-TZVP level.

Furthermore, SP energies are computed by employing the domain-based local pair natural orbital coupled-cluster theory (DLPNO-CCSD(T)), with and without taking into account the ZPE correction energy. We compute the T_1_ diagnostic to determine if the energy evaluation scheme is properly described by a single reference method of the Be_4_B_8_ cluster or contains a multireference character. Our results confirm that the computed T_1_ diagnostic values are below the recommended threshold of 0.02 [122,129] for all low-energy isomers. Hernandez et al. [122] found similar values for the T_1_ descriptor in doped boron clusters. The T_1_ diagnostics and the SP calculations at the DLPNO-CCSD(T) level were performed using the ORCA program suite with TightPNO settings [130].

The chemical bonding was examined using the adaptive natural density partitioning (AdNDP) method [131]. The AdNDP analyzes the first-order reduced density matrix and recovers Lewis bonding (1c–2e or 2c–2e, i.e., lone pairs or two-center two-electron bonds) and delocalized bonding elements (associated with the concept of electron delocalization).

### 2.2. Thermochemistry Properties

The molecular partition function contains all thermodynamic information in a similar way to how the wavefunction contains all the information about the system [33,76], which implies that all the thermodynamic properties of an ensemble of molecules can be derived from this function.

Previous theoretical studies have used the partition function to compute temperature-dependent entropic contributions [132] to a [Fe(pmea)(NCS)2] complex, infrared spectroscopy on an anionic Be_6_B_11_^−^ cluster [33], and rate constants [76]. In this study, the temperature-dependent thermodynamic functions were computed employing the partition function Q, shown in Equation (1), using the rigid rotor, harmonic oscillator, Born–Oppenheimer, ideal gas, and particle-in-a-box approximations.

(1) $$Q\left(T\right)=\underset{i}{\sum }g_{i}e^{-{\Delta E}_{i}/k_{B}T}$$

Here, *g_i_* is the degeneracy factor, *K_B_* is the Boltzmann constant, *T* is the temperature, and −∆*E_i_* is the total energy of a cluster [33,76,133]. We employ Equations (2)–(5) to compute the internal energy (*U*), enthalpy (*H*), and Gibbs energy (*G*) of the Be_4_B_8_ cluster at temperature T. Equations (2)–(5) and the equations to compute entropy contributions (*S*) are the same as those employed in a previous work [33,76] and any standard thermodynamics textbook [133,134].

(2) $$U_{0}=\varepsilon_{0}+ZPE$$

(3) $$U_{T}=U_{0}+\left(E_{Rot}+E_{Trans}+E_{Vib}\right)$$

(4) $$H=U_{T}+RT$$

(5) G=H−ST

Here, *ZPE* is the zero-point energy correction; $\varepsilon_{0}$ is the electronic energy; *E_Rot_* + *E_Trans_* + *E_Vib_* are the contributions to energy due to translation, rotation, and vibration as function of temperature, respectively. In order to compute the probability of the occurrence of one particular cluster in an ensemble of Be_4_B_8_ clusters (Boltzmann ensemble at thermal equilibrium) as a function of temperature, we employed the probability of occurrence [33,76,80,81,135,136,137,138,139,140] given in Equation (6):

(6) $$P\left(T\right)=\frac{e^{-{\beta \Delta G}^{K}}}{\sum e^{-{\beta \Delta G}^{K}}}$$

where $\beta =1/k_{B}T$, $k_{B}$ is the Boltzmann constant, *T* is the temperature, and ΔG*^k^* is the Gibbs free energy of the *k*th isomer. Any molecular properties observed are statistical averages over a Boltzmann ensemble of clusters. For an ensemble of clusters, any property can be computed as an average of all possible conformations [33,138]. Equation (6) is restricted so that the sum of all the probabilities of occurrence at fixed temperature *T*, *P_i_* (*T*), must be equal to 1, according to Equation (7):

(7) $$\overset{n}{\underset{i=1}{\sum }}P\left(T\right)=1$$

In this study, the Boltzmann-weighted *VCD*/*IR* spectrum (*VCD*/*IR_Bolt_*) at temperature *T* is given by Equation (8):

(8) $$VCD/IR_{Bolt}=\sum_{i=1}^{n}\left(VCD_{i}/IR_{i}\right)P_{\left(i\right)}\left(T\right),$$

where *n* is the total number of clusters in the ensemble, *VCD*/*IR_i_* is the *VCD/IR* of the *i*th isomer at temperature *T* = 0, and *P_i_* (*T*) is the probability of the *i*th isomer given by Equation (6). The sum only runs over all achiral, plus, and minus isomers. For achiral structures, the VCD is equal to zero, and there is no contribution to *VCD_Bolt_*. Here, we point out that it is important to take into account the achiral structures due to the probability of a particular chiral cluster changing as a consequence of the *VCD_Bolt_*, in spite of the *VCD* for achiral structures being zero. For the computation of relative populations and *VCD*/*IRBolt* spectra, we used the Boltzmann Optics Full Ader code (BOFA), which is part of the spectroscopy capabilities of the GALGOSON code [33].

## 3. Results and Discussion

### 3.1. The Lowest Energy Structures and Energetics

**Figure 1:**
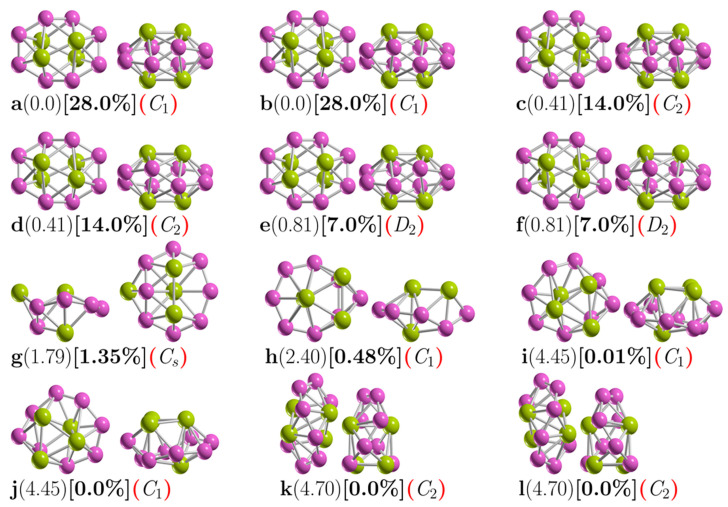
(Color online) Optimized geometries of a neutral Be_4_B_8_ cluster at the PBE0-GD3/def2TZVP level of theory with ZPE correction energy. These are shown in front and side views. The first letter is the isomer label, the relative Gibbs free energies in kcal·mol^−1^ (in round parenthesis) at 298.15 K, the relative population (in square brackets), and the group symmetry point (in red round parenthesis). The structures with labels (**a**,**b**), (**c**,**d**), (**e**,**f**), (**i**,**j**), (**k**,**l**), and (**g**,**h**) are chiral. The purple- and yellow-colored spheres represent the boron and beryllium atoms, respectively [33]. The atomic cartesian coordinates of these isomers are provided in the Supplementary Materials.

Figure 1 shows the low-energy configurations of Be_4_B_8_ clusters optimized at the PBE0-GD3/def2-TZVP level of theory, including ZPE energy correction. The optimized average B–B bond length of the putative chiral global minimum is 1.5867 Å, which is in good agreement with the experimental bond length of 1.57–1.59 Å [141,142] and with the results of other previous DFT calculations [33]. The most frequently recurring motif within the lower-energy isomers of Be_4_B_8_ is a sandwich structure (SSh), in which the boron atoms form a hollow distorted ellipsoid ring with each of the Be–Be dimers capping the top and bottom with C_1_ point group symmetry.

Isomers depicted in Figure 1a,b, listed as i_1_ and i_2_ in Table 1, are enantiomers differing in the orientation of the Be–Be dimers with respect to the boron skeleton. Based on the B–B bond length evolution along the intrinsic reaction coordinate (IRC) path (see videos in the Supplementary Materials) between plus-enantiomers and minus-enantiomers (displayed in Figure S1 of the Supplementary Materials), the shortest B–B bond length is located at the transition state structure. In contrast, the largest B–B bond length is located in the reactant and product points. On the other hand, Figure S2 of the Supplementary Materials shows the distance evolution between Be–Be bond length and (Be–Be) dimers distance; one can see that the largest distance between dimers is located at the transition state, whereas the shortest distance is located at the product and reactant points.

As mentioned above, the B–B interaction does not favor the formation of the lowest-energy enantiomers structures; meanwhile, the Be–Be interaction encourages the lowest-energy structure to be chiral. Here, we infer that the Be–B interaction also favors chiral lowest-energy structures. The Be–Be bond lengths for the six lowest-energy enantiomers are 1.9874, 1.9876, and 1.9881 Å for symmetries C_1_, C_2_, and D_2_, respectively, which is in good agreement with the bond length of the Be–Be in Be_2_B_8_ cluster (1.910 Å) [53].

**Figure 2:**
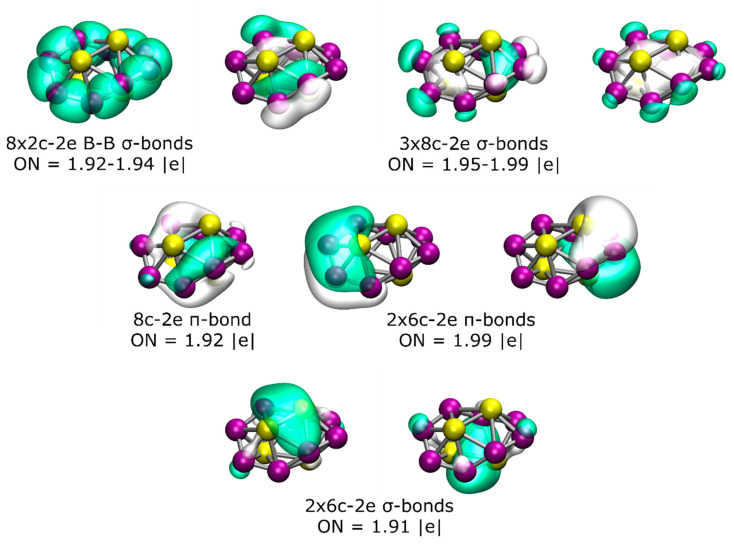
(Color online) Results of the AdNDP analysis of the lowest-energy isomer of the Be_4_B_8_ system.

To gain more insight into the chemical bonding, an AdNDP analysis of the lowest-energy isomer was performed (Figure 2). The AdNDP analysis for this chiral structure revealed the presence of eight 2c-2e B–B σ-bonds with an occupation number (ON) of between 1.92 and 1.94 |e| and three delocalized σ-bonds throughout the B_8_ ring with an ON between 1.95 and 1.99 |e|. Additionally, there are three distorted π-bonds (due to the non-planarity of the structure), one of which is delocalized over all eight boron atoms and the other two involving four boron and two beryllium atoms (one from the top and one from the bottom). Finally, the bonding pattern is completed by two 6c-2e σ-bonds, with the main contribution coming from the interaction between the two Be atoms from the top and bottom, respectively.

The isomers with symmetry C_1_ are the most energetically favorable, with 28% each of the Boltzmann population at 298.15 K. An exhaustive and systematic exploration of the potential energy surface considering triplet states revealed that the lowest triplet ground state lies 13.7 kcal·mol^−1^ above the singlet putative chiral global minimum ground state, which is too far away energetically to be considered. Next, low-energy SSh isomers labeled *i*_3_ and *i*_4_ in Table 1 and depicted in Figure 1c,d lie just 0.41 kcal/mol above the putative global minimum. It is a similar SSh structure to the putative global minimum, except with C_2_ point group symmetry, followed by a slightly higher-energy and similar SSh structure located just 0.81 kcal/mol above the putative minimal structure with D_2_ point group symmetry.

We assert that the unique differences between these chiral structures are due to their different symmetry point groups. The most energetically favorable is the non-symmetry (C_1_) cluster. In addition, these six structures contribute to 98% of the relative population at 298.15 K. The next highest-energy structure, labeled *i*_7_ in Table 1 and depicted in Figure 1g, is located 1.79 kcal/mol above the putative minimum global at 298.15 K with the symmetry C_s_. This is also a sandwich structure formed by a distorted circular ring in which one of the Be–Be dimers caps is in the center of the ring, and the other one is located on one face of the boron circular ring. This structure is achiral, and its probability of occurrence is 1.35% at 298.15 K.

The next chiral isomer lies 2.40 kcal/mol above the putative minimum global with C_1_ symmetry and is labeled *i*_8_ in Table 1 and depicted in Figure 1h. It is also a sandwich-type structure formed by a distorted circular boron ring, with three boron atoms capping one side of the ring and the other Be atom capping the other side. The probability of occurrence of this isomer at 298.15 K is just 0.48%, and its contribution to chiroptical spectroscopies is negligible.

The next two chiral structures lie 4.45 kcal/mol above the putative global minimum with C_1_ symmetries and are labeled *i*_9_ and *i*_10_ in Table 1 and depicted in Figure 1i,j. They are sandwich-type structures formed by a non-planar distorted circular boron ring with three Be atoms capping one side of the boron ring; the other Be atom is located on the other side and in the center of the distorted boron ring. Its Boltzmann probability of occurrence is zero at 298.15 K; thus, at this temperature, its contributions to any chiroptical spectroscopies are negligible.

The following chiral higher-energy structure, with C_2_ point group symmetry, lies 4.70 kcal/mol energy above the putative global minimum. It is a chiral helix-type structure, as depicted in Figure 1k,l. It has four Be atoms located in the center of the boron spiral; this helix structure is similar to those found by previous theoretical works [18,21,33], and its probability of occurrence is negligible at room temperature.

To gain insight into the energy hierarchy of isomers and validate our DFT calculations, relative energies were computed at different levels of theory, and the differences between them are shown in Table 1. Computing energies using different methods yield different results, mainly due to the functional and basis sets employed [33,143], so the energetic ordering changes. Consequently, the probability of occurrence and the molecular properties will change. The first line of Table 1 shows the relative Gibbs free energy computed at PBE0-GD3/def2-TZVP and room temperature. The small relative Gibbs free energy (0.41 and 0.81 kcal/mol) differences among the six enantiomer structures *i_1_* to *i_6_* in Table 1 are caused by the rotational entropy being a function of the symmetry number, which in turn depends on the point group symmetry. An increase/decrease in the value of rotational entropy will change the Gibbs free energy. The Gibbs free energy computed with and without symmetry will differ by a factor of RTlnσ. Here, *R* is the universal gas constant, *T* is the temperature, and σ is the symmetry number.

**Figure 3:**
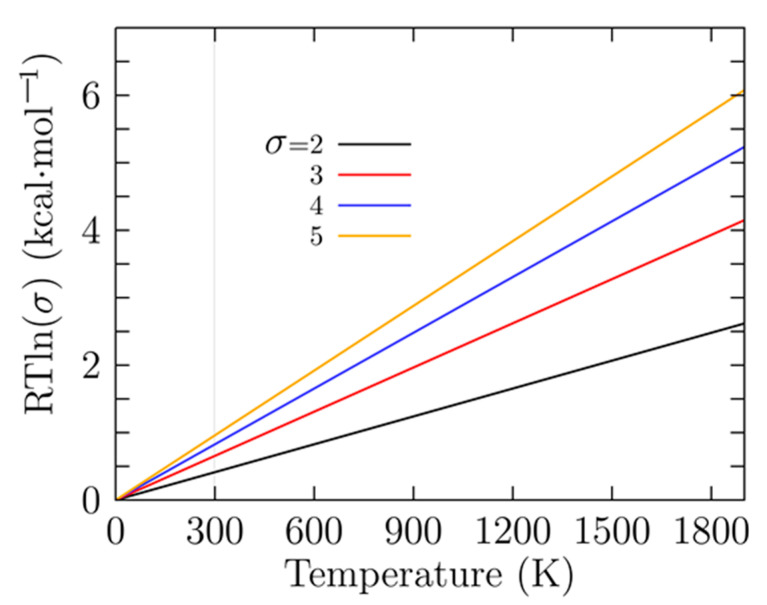
(Color online) The difference in the rotational entropy computed with and without the symmetries is given by a factor of RTlnσ in kcal/mol; in this factor, *R* is the universal gas constant, *T* is the temperature, and σ is the symmetry number. The factor is similar to the enantioselectivity [144].

Figure 3 shows the factor RTlnσ for temperatures ranging from 0 to 1900 K and for different symmetry number values (σ=2,3,4,5). A closer analysis of Figure 2 shows that at room temperature RTlnσ is 0.41 kcal/mol with σ=2 and 0.81 kcal/mol with σ=4, which is in agreement with the values shown in the first line of Table 1. As the temperature increases, the energy differences between the RTlnσ factors become larger. These small relative Gibbs free energies are responsible for the different values of probability of occurrence at low temperatures for similar isomers with different point group symmetries. This strongly suggests that there must be atomic clusters with low and high symmetries in the Boltzmann ensemble in order to compute the molecular properties correctly.

The second line in Table 1 shows the single-point (SP) relative energies computed at the CCSD(T) [145]. The energetic ordering of the isomers listed in the first line of Table 1 almost follows the trend of energetic ordering at the SP CCSD(T) level; notice that only the achiral isomers labeled *i*_7_ and *i*_8_ in Table 1 are interchanged in energetic ordering. The third line in Table 1 shows the single-point relative energies computed at the CCSD(T) [145]/def2-TZVP//PBE0-GD3/def2-TZVP; the energetic ordering is similar to that for pure CCSD(T) energy. The DLPNO-CCSD(T) relative energies, with and without ZPE correction, are shown in lines four and five of Table 1; the first follows the trend of pure CCSD(T) energy, while the second—the ZPE value—changes the isomer *i_7_* in Table 1 to be the putative global minimum. Here, we can say that the ZPE energy inclusion is essential in distributing isomers and molecular properties.

The sixth and seventh lines of Table 1 show the electronic energy with and without ZPE correction, and both of them follow the trend of the Gibbs free energy given in line number one. Line number eight in Table 1 shows the point group symmetry for each isomer. The T_1_ diagnostics for each isomer are shown in line nine of Table 1; all of them are lower than the recommended value of 0.02 [122,145], suggesting that the systems are appropriately characterized.

### 3.2. Structures and Stability at Finite Temperature

**Figure 4:**
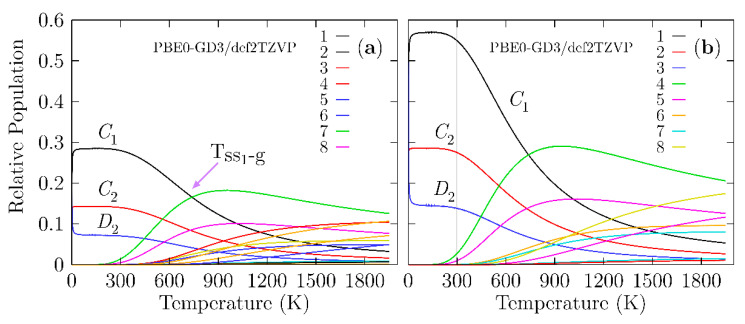
(Color online) (**a**) The relative population (probability of occurrence) for temperatures ranging from 20 to 1900 K at the PBE0-GD3/def2-TZVP level of theory considering that the Boltzmann ensemble is composed of achiral structures and an equal mixture of P and M enantiomers, which implies a Boltzmann racemic ensemble. (**b**) The relative population for temperatures ranging from 20 to 1900 K at the PBE0-GD3/def2-TZVP level of theory, taking into account only the achiral structures and M enantiomers, suggestive of a Boltzmann pure ensemble of only one enantiomer. In (**a**), the transition solid–solid point (T_ss1-g_) is located at 739 K with a 16.6% probability, while in (**b**), the T_ss1-g_ is located at 739 K with a 27% probability. The lowest-symmetry C_1_ strongly dominates at temperatures from 20 to 739 K due to the rotational entropy that is a function of the point group symmetry.

As previously stated, the determination of the structure is the first step in studying any property of a material. We have to consider that an observed molecular property in a Boltzmann ensemble is a weighted sum of all the individual contributions of each isomer that form the ensemble. At 0 K, the electronic energy plus the zero-point energy determines the putative global minimum and all nearby low-energy structures (PGMLES). At temperatures larger than 0 K, the Gibbs free energy defines the PGMLES. Figure 4 shows the probability of occurrence for each particular chiral and achiral Be_4_B_8_ isomer for temperatures ranging from 20 to 1900 K.

In Figure 4a, the probability of occurrence is displayed, taking into account the M, P, and achiral structures shown, which implies that the percent enantiomeric excess is zero (Boltzmann racemic ensemble). Figure 4b shows the probability of occurrence when only taking into account the M enantiomer and achiral structures, which implies that the percent enantiomeric excess is 100%. Then, the ensemble is a pure Boltzmann ensemble of only one enantiomer. There is a significant difference in the probability of occurrence between the two panels. In Figure 4a, we consider the P and M structures, and both structures possess the same probability of occurrence in all ranges of temperature. All the probabilities of occurrence (chiral) shown in Figure 4b are approximately two times the probability of occurrence (chiral) shown in Figure 4a.

A closer examination of Figure 4a shows that in the temperature range from 20 to 300 K, all molecular properties are dominated by the chiral structure depicted in Figure 1a,b because its probability of occurrence is almost constant. We point out that in this range of temperature, the C_1_, C_2_, and D_2_ symmetries strongly dominate, with probabilities of occurrence of 28, 14, and 7%, respectively. These different probabilities of occurrence for the same structure with only different symmetries occur due to rotational entropy, which is also responsible for the slight energy differences shown in Table 1. In turn, this is the reason for the differences in the probability. At temperatures above 300 K, the probability of occurrence of the putative global minimum at cold temperatures, depicted in a solid black line, decay exponentially up to 1900 K.

The dominant transformation solid–solid point (T_ss1-g_) is located at 739 K with a 16.6% probability. At this point, there is a co-existence of chiral structures and achiral structures, as shown in Figure 1a,g. Above this point, the achiral structure (Figure 1g) becomes dominant. Its probability of occurrence is depicted by the solid green line in Figure 4a and starts to increase at almost room temperature. The second transformation solid–solid point located at 1017 K with a 10% probability also has a chiral putative global minimum with symmetry C_1_ and an achiral structure (Figure 1h) located at 2.40 kcal/mol Gibbs free energy at 298.15 K above the putative global minimum. Figure 3 shows the computed probability of occurrence considering the percent of enantiomeric excess to be one hundred percent, which is suggestive of a pure Boltzmann ensemble of only one enantiomer.

With the aim of computing the Boltzmann VCD/IR-weighted spectra as a function of temperature, we used the relative population shown in Figure 4a. The analysis of the probability of occurrence leads us to an interesting observation: the individual putative global minimum strongly dominates the VCD/IR at temperatures ranging from 20 to 1240 K. The achiral structures have zero contribution to VCD in hot temperatures. The probability of occurrence is dependent on the functional and basis set employed as a result of the energies computed using different methods [143]. Figure S3 in the Supplementary Materials shows the relative population computed at the TPSS [146]-GD3/def2-TZVP level of theory.

At cold temperatures, the chiral structure with symmetry C_1_, as depicted in Figure 1a,b, strongly dominates. At hot temperatures, the dominant structure is a chiral helix-type structure depicted in Figure 1k,l and is located 4.70 kcal/mol (in terms of Gibbs free energy) above the putative global minimum. Additionally, at SP CCSD(T) level, it is located at a relatively high energy above the global minimum. The relative population employing the TPSS functional does not follow the energetic ordering trend, as it does at the CCSD(T) level of theory. The above discussion proves that the probability of occurrence is sensitive to the level of theory.

### 3.3. Enantiomerization Energy Barrier at Finite Temperature

The process in which a pair of chiral molecules or enantiomers undergo the conversion of one enantiomer to another is referred to as enantiomerization. Enantiomers each have the same free energy and equal probability of occurrence, as shown in Figure 4. The extent of the interconversion of enantiomers depends on the energy barriers to enantiomerization. In addition, this energy barrier determines if an enantiomer can be resolved at temperature T and defines its configurational stability. There are cases where the enantiomerization is undesirable; for example, many drugs are related to chirality, and frequently, only one of the enantiomers shows the desired effect, while the other shows undesirable effects. Chiral molecules with a high charge-carrier mobility and fluorescence quantum yield need high-energy barriers for enantiomerization [147].

**Figure 5:**
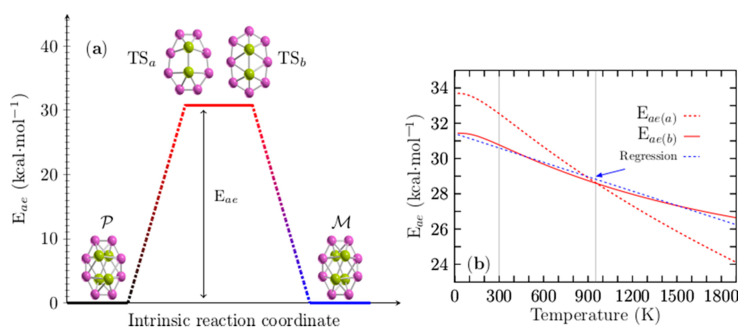
(Color online) In panel (**a**), there are two transition states close in energy, TS_a_ and TS_b_, and two possible intrinsic reaction coordinates (see videos in the Supplementary Materials): Route a: computed activation energy (E_ae(a)_) of 32.5 kcal·mol^−1^. Route b: computed activation energy E_ae(b)_ of 30.77 kcal·mol^−1^ between P and M chiral Be_4_B_8_ clusters at room temperature. The energy barriers for interconversion are equal to each other at temperature T (temperature barrier–barrier T_bb_ point), which implies that the rates of reaction of both mechanisms are equal. For the case of chiral Be_4_B_8_, T_bb_ is located at 954 K. In panel (**b**), E_ae(a)_ is depicted as a red dashed line, and E_ae(b)_ is depicted as a red solid line for temperatures ranging from 20 to 1900 K. At 954 K, the blue-dashed line in panel (**b**) represents a linear regression in the computed data of the energy barrier E_ae(b)_, depicted as a red solid line in panel (**b**); its correlation coefficient is −0.9925.

Figure 5a shows the computed enantiomerization energy barrier (energy activation (E_ae_) or Gibbs free activation energy (∆G‡)) of the pair of enantiomers, P and M, of the Be_4_B_8_ cluster, which has only a single step for two mechanisms of reaction and for which the energy barriers are energetically similar. The transition states (TS_a_, TS_b_) depicted in Figure 5a are achiral sandwich-type structures in which the boron atoms form a planar ring with each of the Be–Be dimers capping the top and bottom; they are aligned parallel to the major axis of the boron ellipse. The main difference between them is a shift in the ring position concerning the Be–Be dimers. The energy barriers related to TS_a_ and TS_b_ are 32.50 and 30.77 kcal/mol, respectively, which indicates that Be_4_B_8_ enantiomers are stable at room temperature. These energy barrier heights are similar to those computed for the Au_38_ (SR)24 [148] clusters that lie in the range of 29.9 to 34.5 kcal/mol. The energies of enantiomerization, E_ea(a)_ and E_ea(b)_, corresponding to TS_a_ and TS_b_ for temperatures ranging from 20 to 1900 K, are displayed in Figure 5b. E_ea(a)_ is depicted as a red dashed line, whereas E_ea(b)_ is depicted as a red solid line.

The analysis of the results leads to an interesting observation: in Figure 5b, one can see that there is a barrier–barrier temperature point (T_bb_) located at 954 K where the energy barriers of both mechanisms are equal. At T_bb_, the probability that the reaction will take one path or another is 50/50%, which implies that the rates of reaction for both reaction mechanisms are equal to each other. Below a temperature of 954 K, reaction path b (TS_b_) is more favorable than reaction path a (TS_a_), and vice versa for temperatures above 954 K.

E_ea(b)_ decreases linearly in the temperature range from 200 to 740 K. Below 200 K and in temperatures ranging from 740 to 1900 K, the energy barrier behavior is non-linear. To make it clearer, a blue dashed line is depicted in Figure S4 of the Supplementary Materials, overlapping the energy barrier in the temperature range from 200 to 740 K. Equation (9) was found using linear regression, with the correlation coefficients −0.9925 of the energy barrier depicted as a red solid line in Figure 5b.

(9) ΔG=31.42−0.00271188T.

In Equation (9), T is the temperature, and it describes approximately the energy barrier in all ranges of temperature. It is depicted in the blue dashed line of Figure 5b. Evaluating Equation (9) with T = 298.15 K gives 30.59 kcal/mol, which is very close to the computed value of 30.77 kcal/mol. The first term of Equation (9) is enthalpy and the second one is the entropic term. The computed values of ΔG, ΔH, and ΔS and the percentage of contribution of ΔS to the energy barrier are summarized in Table 2 for some temperature values.

The analysis of results shown in Table 2 indicates that the enthalpy term is too large compared with the entropic term as shown in columns 3 and 4 of Table 2, respectively, and it is evaluated for the range temperatures given in column 1 of Table 2. Column five of Table 2 shows the percentage at which the energy barrier decreases as a function of temperature and due to the entropic term, considering the energy barrier computed at T = 0 K as the reference. Notably, the composition of the energy barrier is enthalpic and too high in all ranges of temperature. We concluded that the interconversion between enantiomers is thermodynamically unfavorable in all ranges of temperature based on our computations. At high temperatures, the energy barrier is still too high, and the most significant entropic contribution does not exceed 15.53%.

### 3.4. Height of the Energy Barrier between Chiral and Achiral Structures at Finite Temperature

**Figure 6:**
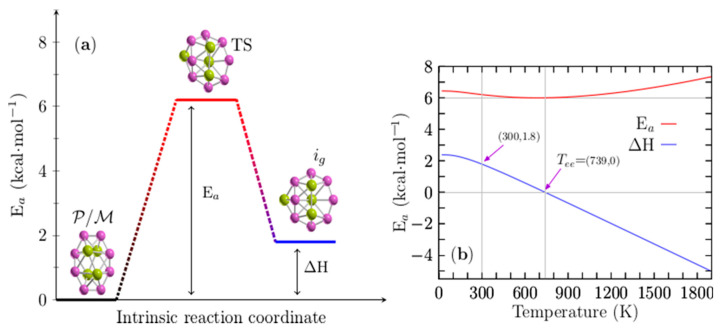
(Color online) (**a**) The height of the energy barrier for interconversion (Ea) is 6.20 kcal·mol^−1^ between P (plus) or M (minus) chiral and achiral structures of Be_4_B_8_ clusters at room temperature. (**b**) The E_a_ is depicted as a solid red line, and the enthalpy of formation (∆H) is depicted as a blue solid line for temperatures ranging from 20 to 1900 K. The ∆H is zero at T = 739 K and 1.8 kcal/mol at T = 300 K. Notice that the E_a_ has a minimum located at 739 K.

Figure 6a displays the height of the energy barrier interconversion at room temperature between the chiral P/M structures shown in Figure 1a,b and the achiral structure depicted in Figure 1g. Remarkably, these structures coexist at the dominant solid–solid transformation point located at 739 K; according to the probability of occurrence at hot temperatures, the achiral structure is the putative global minimum. In addition, the endergonic to exergonic temperature point, T_ee_, is defined here as the temperature at which the reaction type changes from endergonic to exergonic. In this Be_4_B_8_ cluster, it coincides with the solid–solid transformation point.

When these two points coincide, at least two structures coexist, and there is a change in the type of reaction from endergonic to exergonic or vice versa at temperature T. For the interconversion between these structures, the height of the energy barrier at room temperature is 6.20 kcal/mol, and the enthalpy of the formation (AH) is 1.8 kcal/mol. The TS is depicted in Figure 6a; it is also a sandwich-type structure formed by a distorted circular ring in which the Be–Be dimers cap each face of the ring. It has a similar structure to that of isomer i_7_ depicted in Figure 1g. Figure 6b shows the height of the energy barrier for the chiral and achiral structures depicted in a solid red line; the enthalpy of formation (∆H) for the same structures is depicted in a solid blue line for temperatures ranging from 20 to 1900 K. An analysis of ∆H in Figure 6b shows that the reaction process is endothermic for temperatures ranging from 20 to 739 K because the ∆H is positive. At a temperature of 739 K the ∆H is zero, which implies that chiral structures with C_1_ symmetry and achiral (i_7_) structures with C_1_ symmetry coexist.

The above discussion is in good agreement with the computed point T_ss−1_ located at 739 K, as displayed in Figure 4a. According to the probability of occurrence, at this point, the chiral and achiral structures coexist. Additionally, at this temperature point, the height of the energy barrier, depicted as a red solid line in Figure 6b, has a minimum value of 6.0 kcal/mol. At temperatures above 739 K, the reaction process is exothermic due to the fact that the ∆H is negative, and the height of the energy barrier slowly increases. 

A more detailed analysis of the results leads to several observations. The reaction process is endothermic up to 739 K, which implies the absorption of energy, and chiral structures strongly dominate as the putative global minimum. At temperatures of 739 K, chiral and achiral structures coexist. At temperatures above 739 K, the reaction process is exothermic, and the non-chiral structures weakly dominate as the putative global minimum. Based on the ∆H behavior in all ranges of temperature, we suggest that the reaction is an entropic-driven process due to the fact that the type of reaction changes from endothermic to exothermic as the temperature increases.

### 3.5. VCD and IR Spectra

**Figure 7:**
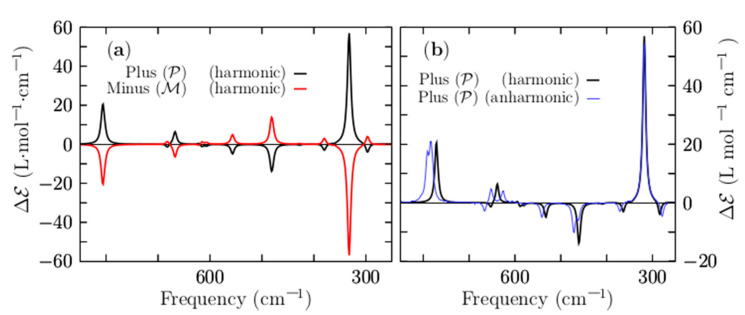
(Color online) (**a**) A comparison of the VCD spectrum for frequencies ranging from 850 to 250 cm^−1^ for the lowest-energy P and M enantiomers. (**b**) A comparison of the VCD harmonic spectrum and VCD anharmonic spectrum to estimate the importance of anharmonicities in the Be_4_B_8_ chiral cluster. The anharmonic vibrational spectrum is depicted as a solid blue line; the harmonic vibrational spectrum is depicted as a solid black line for the lowest-energy enantiomer P. A scale factor of 0.96 is applied to shift the harmonic spectrum to overlay the anharmonic spectrum. The spectra are the results of a convolution of a Lorentzian shape profile with an FWHM of 20 cm^−1^ on the computed discrete frequency intensities.

Figure 7a shows a comparison of the VCD harmonic spectra, corresponding to the P and M lowest-energy structures, depicted in solid black and red lines, respectively. They show a mirror image relationship, thereby ensuring that the two structures are non-superposable. The computed VCD spectrum (P structure) is characterized by five main peaks located at frequencies of 330, 481, 802, 1062, and 1208 cm^−1^, respectively. The largest peak with a positive intensity is located at 330 cm^−1^, and it corresponds to the stretching of the two Be–Be dimers capping the distorted boron ring. Next, the transition located at 481 cm^−1^ is the largest negative and is attributed to the bending of the boron distorted ring in a kind of breathing motion.

The peaks located in the region of 1208–1062 cm^−1^ correspond to ring boron stretching. The harmonic approximation works if the potential energy is parabolic and fails [149], as the temperature increases due to anharmonic effects [149]. Under harmonic approximation, strongly anharmonic systems cannot be well described [150,151]. For high temperatures above 0.7 or 0.8 times the melting temperature, explicit anharmonic contributions become relevant [152]; in addition, we have to consider whether or not the cluster is highly anharmonic. To estimate the importance of the anharmonicities of the Be_4_B_8_ chiral cluster, we show in Figure 7b the anharmonic VCD spectra, which are depicted as a solid blue line. For ease of comparison, this is overlaid with the harmonic vibrational spectrum, which is depicted as a solid black line. Both of them were computed for the P lowest-energy structure employing the Gaussian 09 code [120].

A shifting factor of 0.96 was applied to shift the harmonic spectrum to overlay the anharmonic spectrum. We found that the frequency shift was 14 cm^−1^ towards high frequency. A comparison of the two spectra, as displayed in Figure 7b, shows that the computed harmonic and anharmonic spectra are in very good agreement. In fact, most of the peaks are correctly computed by employing the harmonic approximation. In the low range of energy, the harmonic peaks and anharmonic peaks agree well; however, there are slight discrepancies in the region of 1200–1100 cm^−1^. In this study, the computations of the thermodynamic properties and VCD spectra using harmonic approximation yielded sufficiently reliable results to describe the non-strongly anharmonic Be_4_B_8_ chiral cluster.

Additionally, Figure S5 in the Supplementary Materials shows the IR spectra computed using harmonic and anharmonic approximations. The IR harmonic spectrum is depicted as a solid black line, whereas the IR anharmonic spectrum is depicted as a solid red line. A shifting factor of 0.96 is applied to impose the IR harmonic spectrum over the IR anharmonic spectrum. Comparing these spectra, it can be seen that they match over a large range of frequencies. Therefore, we infer that the IR spectra found using harmonic approximation yield valid results.

Regarding the temperature-dependent VCD spectra, the Boltzmann-weighted overlapping needed to yield a total VCD spectrum at all ranges of temperatures is zero, because the Boltzmann ensemble is composed of achiral structures and an equal mixture of both P and M enantiomers, which implies that the percent enantiomeric excess is zero. Therefore, the Boltzmann ensemble is racemic. Any chiroptical response in the Be_4_B_8_ cluster must be null. The exhaustive exploration of potential and free energy surfaces revealed that there are 22 isomers within an energy range of 0 to 9.2 kcal/mol, six of which are chiral structures with symmetries of C_1_, C_2_, and D_2_, and these were within 1 kcal/mol. These structures compose 98% of the relative population at room temperature.

**Figure 8:**
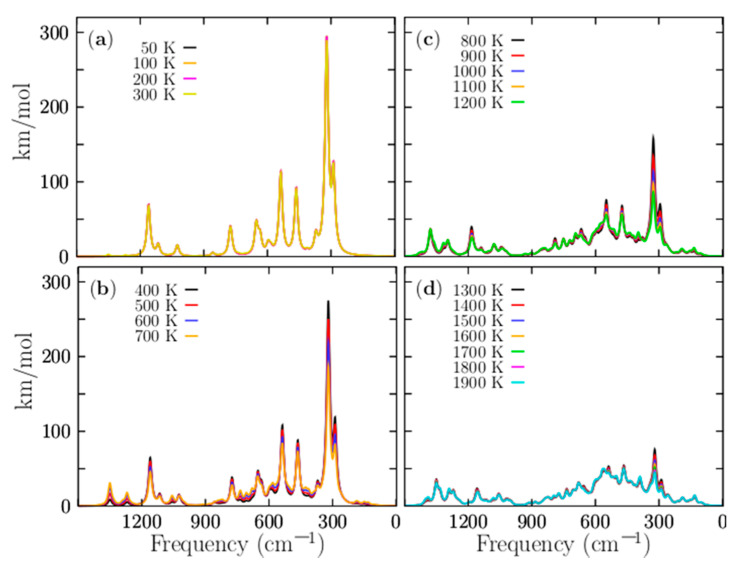
(Color online) The temperature-dependent IR Boltzmann-weighted spectra of the P Be_4_B_8_ ensemble computed at the PBE0-D3/def2-TZVP level of theory computed in frequency range of 1500 to 1 cm^−1^. Three similar chiral conformers with C_1_, C_2_, and D_2_ symmetries, which correspond to 96.3% of the Boltzmann distribution, strongly dominate the IR Boltzmann spectra-weighted temperatures from 0 to 1200 K. The IR Boltzmann-weighted spectra for different temperature ranges: (**a**) 50–300 K, (**b**) 400–700, (**c**) 800–1200, and (**d**) 1300–1900 K. At temperatures below 300 K, the amplitudes of the spectra are constant, which is in good agreement with the relative population. At temperatures above 300 K, the magnitude of the spectrum decreases exponentially until 1200 K. The spectra were computed employing Lorentzian with half widths at half maxima of 20 cm^−1^. The computed frequencies were multiplied by a scaling factor of 0.96. The images are a little blurry because of the overlapping of several signals.

With the aim to compute the Boltzmann-weighted IR spectra, structures which only differ in the symmetry group have to be taken into account. Figure 8 shows the IR spectra. For temperatures ranging from 50 to 1900 K, the IR spectrum is composed of five peaks. The most considerable peak intensity is located at 330 cm^−1^, and it still keeps strongly dominating in temperatures ranging from 20 to 700 K. This mode corresponds to the alternating stretching of the two Be–Be dimers capping the distorted boron ring, and it is a mode that contributes to interconversion between P and M structures. There are another four modes with smaller intensities that also benefit the interconversion between P and M structure; they are located at 300, 333, and 361 cm^−1^, respectively. So, at cold temperatures, all vibration modes located ranging from 300 to 361 cm^−1^ are related, in one way, with the stretching of the beryllium atoms. In contrast, at hot temperatures, those vibrational modes tend to be negligible. The other modes are related to the compression/expansion of the boron ring. Figure 8a displays the IR spectra for temperatures ranging from 50 to 300 K; in this range, the IR spectra are strongly dominated by the lowest energy pair of enantiomers with C_1_ symmetry, and further, the IR intensities remain constant in this range of temperatures.

The above mentioned agrees with the relative population depicted in Figure 4a where the probability of occurrence of the pair of enantiomers with symmetry C_1_ strongly dominates. We have to consider that the contribution to the IR spectra of the four enantiomers with C_2_ and D_2_ symmetries for temperatures ranging from 20 to 300 K is equal to the IR spectrum with symmetry C_1_, and there is no presence of other structures. Therefore, at room temperature, all molecular properties, except for the chiral properties, are attributable to the lowest-energy pair of enantiomers, depicted in Figure 1a,b.

Figure 8b shows the IR spectra for temperatures ranging from 400 to 700 K. The IR intensities start with exponential decay, in agreement with the probability of the occurrence of the lowest pair of enantiomers of Figure 4a. There is a little contribution from other isomers, but not enough to alter the IR spectrum. Therefore, the shape of the IR spectrum remains equal to the IR spectrum at cold temperatures. The IR spectra for temperatures ranging from 800 to 1200 K are shown in Figure 8c. The largest contribution of a particular isomer is less than 17%, thus, the largest peak of the IR spectra tends to be neglected. Figure 8d shows that the IR spectra is almost null, thus, at hot temperatures, the IR spectra are neglected, because almost all the contributions of the isomers to the IR spectrum are around 10%.

### 3.6. Molecular Dynamics

We performed Born–Oppenheimer molecular dynamics employing the deMon2K program [153] (deMon2k v. 6.01, Cinvestav, Mexico City, Mexico, 2011) at different temperatures (1100, 1200, and 1500 K), aiming to gain insight into the dynamical behavior of the Be_4_B_8_ cluster (see videos in the Supplementary Materials). The simulation time was 25 ps with a step size of 1 fs. For the Be_4_B_8_ cluster, we found a dissociation phenomenon when the temperature was higher than 1200 K; at 1500 K, the dissociation process was stronger, while at 1100 K, there was no dissociation. At temperature T, a cluster dissociates, and the melting point temperature is lower than the temperature of dissociation [33,154].

## 4. Conclusions

We have estimated the probability of occurrence of each isomer of the Be_4_B_8_ cluster under the framework of nanothermodynamics. Our findings showed that the putative global minimum of Be_4_B_8_ is a chiral structure and, at cold temperatures, is strongly dominant.

We have analyzed the effects of the point group symmetry on the Gibbs free energy and on the probabilities as a function of temperature. Additionally, we demonstrated that the slight relative Gibbs free energy differences of 0.41 and 0.81 kcal/mol at room temperature between different symmetries are due to rotational entropy.

Furthermore, the relative population shows that three structures co-exist at a temperature of 739 K, and a solid–solid transformation occurs. Investigation on the solid–solid transformation between P and M structures reveals that the two enantiomerization energies are high. Additionally, there are two different reaction mechanism very close in energy; at the temperature of 954 K, the enantiomerization energies barriers are equal to each other.

The temperature-dependent solid–solid transformation between P/M and g structures that co-exist at 739 K reveals that the chemical reaction is endothermic at cold temperatures, whereas at hot temperatures, it is exothermic. The clear temperature dependence of the Boltzmann-weighted spectra are modulated just by probabilities of the putative low-energy isomers at temperatures ranging from 20 to 739 K. At temperatures above 739 K; the spectra decay strongly. In contrast, at temperatures above 1200 K, the spectra are almost null.

As future work, an extension of this methodology to systems with periodic boundary conditions will be considered.

## Figures and Tables

**Table 1:** Single-point relative energy calculations of the low-energy structures from *i*_1_ to *i*_10_ at different levels of theory: coupled cluster single-double and perturbative triple (CCSD(*T*)), CCSD(*T*) with zero-point energy ($\mathrm{CCSD}\left(T\right){+\varepsilon }_{\mathrm{ZPE}}$, CCSD(*T*)) employing the domain-based local pair natural orbital coupled-cluster theory (DLPNO-CCSD(*T*)), with TightPNO setting, and with $\varepsilon_{\mathrm{ZPE}}$ ($\mathrm{DLPNO}-\mathrm{CCSD}\left(T\right){+\varepsilon }_{\mathrm{ZPE}}$). Gibbs free energy (ΔG) at 298.15 K, electronic energy with $\varepsilon_{\mathrm{ZPE}}$ ($\varepsilon_{0}{+\varepsilon }_{\mathrm{ZPE}}$), electronic energy ($\varepsilon_{0}$), point group symmetry, and *T_1_* diagnostic. All relative energies are given in kcal·mol^−1^.

Level	i_1_	i_2_	i_3_	i_4_	i_5_	i_6_	i_6_	i_8_	i_9_	i_10_
ΔG	0.0	0.0	0.41	0.41	0.81	0.81	1.79	2.40	4.45	4.45
CCSD(T)	0.0	0.0	0.0	0.0	0.0	0.0	3.61	3.38	5.38	5.38
$\mathrm{CCSD}\left(T\right){+\varepsilon }_{\mathrm{ZPE}}$	0.0	0.0	0.0	0.0	0.0	0.0	2.71	2.51	4.51	4.51
DLPNO-CCSD(T)	0.0	0.0	0.0	0.0	0.0	0.0	0.75	1.37	5.0	5.0
$\mathrm{DLPNO}-\mathrm{CCSD}\left(T\right){+\varepsilon }_{\mathrm{ZPE}}$	0.0	0.0	0.0	0.0	0.0	0.0	−0.20	0.50	4.10	4.10
$\varepsilon_{0}+\varepsilon_{\mathrm{ZPE}}$	0.0	0.0	0.0	0.0	0.0	0.0	2.38	2.80	5.03	5.03
$\varepsilon_{0}$	0.0	0.0	0.0	0.0	0.0	0.0	3.28	3.68	5.90	3.28
Point Group Symmetry	C_1_	C_1_	C_2_	C_2_	C_1_	C_1_	C_1_	C_1_	C_2_	C_2_
$T_{1}$	0.019	0.019	0.019	0.019	0.019	0.019	0.019	0.019	0.019	0.019

**Table 2:** Approximate energy barrier, enthalpy, entropy terms, and percentage at which the energy barrier decreases, considering the energy barrier computed at *T* = 0 as a reference.

Temperature (K)	ΔG	ΔH	ΔST	% (Decrease)
0	33.42	31.42	0.0	0.0
300	30.60	31.42	0.81	2.58
600	29.79	31.42	1.62	5.17
900	28.97	31.42	2.44	7.76
1200	28.16	31.42	3.25	10.35
1500	27.35	31.42	4.06	12.94
1800	26.53	31.42	4.88	15.53

## Data Availability

Data is contained within the article.

## Supplementary Materials

The following are available online: The xyz atomic coordinates of the optimized Be_4_B_8_ cluster at the PBE0-D3/def2-TZVP/Freq level of theory. The video of IRC for the interconversion between P and M enantiomers through route A B4B8 route_a_IRC.mp4, and through route B Be4B8 route_b_IRC.mp4. The video of IRC for P/M chiral structure to the first achiral structure Be4B8_irc_from_chiral_to_achiral.mp4 The video of molecular dynamics at 1100, 1200, and 1500 K, Be4B8_MD_1100K.mp4, Be4B8_MD_1200K.mp4, and Be4B8_MD_1500K.mp4, respectively. Figure S1. Panel (a) shows the bond length evolution of the Be-Be dimer that is capping one side of the distorted ring boron along with the IRC of the chiral Be4 B8 cluster. Panel (b) shows the evolution of distance between the two dimers that are capping the distorted ring boron along with the IRC of the chiral Be4 B8 cluster. In panel (a), the minimum Be-Be bond length is located at TS state with a value of 1.9416 Å, and the maximum value is 1.9862 Å that corresponds to one of the putative global minima. The largest rate of decreasing/increasing bond length of Be-Be dimer is happening when the reaction start/end, before or after the maximum force point. (see video IRC), Figure S2. Panel (a) shows the bond length evolution of the B-B bond length along with the IRC of the chiral to achiral Be4B8 cluster. Panel (b) shows the evolution of distance between the two di-mers that are capping the distorted ring boron along the IRC of the chiral to achiral Be4B8 cluster, Figure S3. We show a straight line in the blue dashed line overlapping the energy barrier for en-antiomers in the temperature range 200 to 740 K, Figure S4. Probability occurrence of each isomer computed employing TSPP functional with the def2TZVP basis set, taking into account version three of Grimme’s dispersion as it is implemented in Gaussian code. The relative energies between two isomers vary considerably with the function-al use. This will affect the temperature-dependent Boltzmann factors computed for each isomer and, therefore, the relative population change, as shown in Figure. Employing TPSS functional, the TSS point is located at 542 K on a temperature scale compared with the TSS point located at 739 K found employing PBE0 functional, Figure S5. We show a comparison between IR Harmonic vs. IR Anharmonic spectra. IR-Harmonic spectrum was scaled by 0.96 to overlap the IR Anharmonic spectrum. The full width at half maximum (FWHM) employed is 20 cm^−1^.

## Sample Availability

Samples of the compounds are not available from the authors.

## Abbreviations

DFT	Density functional theory
CCSD(T)	Coupled cluster single-double and perturbative triple
DLPNO-CCSD(T)	Domain-based local pair natural orbital coupled-cluster theory
ZPE	Zero-point energy
VCD	Vibrational circular dichroism
IR	Vibrational infrared spectrum
BOFA	Boltzmann Optics Full Ader code (module nanothermodynamics)
GALGOSON	Global genetic algorithm of University of Sonora
SSh	Sandwich structure hollow
AdNDP	Adaptive natural density partitioning
