# Supplementary material for: Theoretical Prediction of Structures, Vibrational Circular Dichroism, and Infrared Spectra of Chiral Be4B8 Cluster at Different Temperatures
Source: Molecules. 2021 Jun 28;26(13):3953. doi: 10.3390/molecules26133953 (PMC8271876; doi:10.3390/molecules26133953)
Supplement: Supplementary file 1 [file molecules-26-03953-s001.zip › molecules-1251824-Supplementary File.pdf]

molecules

MDPI

Supplementary Materials

Theoretical Prediction of Structures, Vibrational Circular Dichroism, and Infrared Spectra of Chiral Be_4B_8 Cluster at Different Temperatures

Carlos Emiliano Buelna-García [1,2], Eduardo Robles-Chaparro [3], Tristan Parra-Arellano [3], Jesus Manuel Quiroz-Castillo [1], Teresa del-Castillo-Castro [1], Gerardo Martínez-Guajardo [4], Cesar Castillo-Quevedo [5], Aned de-León-Flores [3], Gilberto Anzueto-Sánchez [6], Martha Fabiola Martin-del-Campo-Solis [5], Ana Maria Mendoza-Wilson [7], Alejandro Vásquez-Espinal [8] and Jose Luis Cabellos [9,*]

1. Departamento de Investigación en Polímeros y Materiales, Edificio 3G, Universidad de Sonora, Hermosillo 83000, Sonora, Mexico; a209205768@unison.mx (C.E.B.-G.); jesus.quiroz@unison.mx (J.M.Q.-C.); teresa.delcastillo@unison.mx (T.d.-C.-C.)
2. Organización Científica y Tecnológica del Desierto, 83150 Sonora, Mexico; a209205768@unison.mx (C.E.B.-G.)
3. Departamento de Ciencias Químico Biologicas, Edificio 5A, Universidad de Sonora, Hermosillo 83000, Sonora, Mexico; a214201767@unison.mx (E.R.-C.); a215207594@unison.mx (T.P.-A.); aned.deleon@unison.mx (A.d.-L.-F.)
4. Unidad Académica de Ciencias Químicas, Área de Ciencias de la Salud, Universidad Autónomade Zacatecas, Km. 6 carretera Zacatecas-Guadalajara s/n, Ejido La Escondida C. P., Zacatecas 98160, Zacatecas, Mexico; germtzguajardo@uaz.edu.mx
5. Departamento de Fundamentos del Conocimiento, Centro Universitario del Norte, Universidad de Guadalajara, Carretera Federal No. 23, Km. 191, C.P., Colotlán 46200, Jalisco, Mexico; castillo.quevedo@cunorte.udg.mx (C.C.Q.); mfmartindelcampo@cunorte.udg.mx (M.F.M.-d.-C.-S.)
6. Centro de Investigaciones en Óptica, A.C., León 37150, Guanajuato, Mexico; gilberto.anzueto@cio.mx
7. Coordinación de Tecnología de Alimentos de Origen Vegetal, CIAD, A.C., Carretera Gustavo Enrique Astiazarán Rosas, No. 46, Hermosillo 83304, Sonora, Mexico; mwilson@ciad.mx
8. Computational and Theoretical Chemistry Group Departamento de Ciencias Químicas, Facultad de Ciencias Exactas, Universidad Andres Bello, Republica 498, 8370035 Santiago, Chile; a.vasquezespinal@uandresbello.edu
9. Departamento de Investigación en Física, Edificio 3M, Universidad de Sonora, Hermosillo 83000, Sonora, Mexico; jose.cabellos@unison.mx

* Correspondence: jose.cabellos@unison.mx

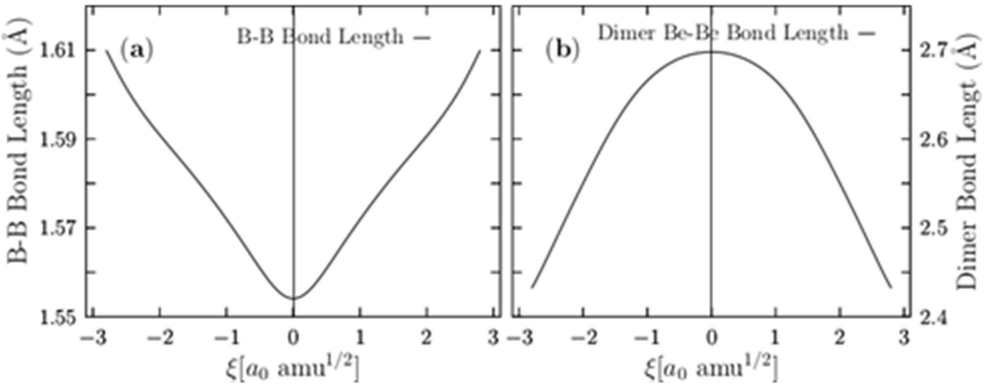

Figure S1. Panel (**a**) shows the bond length evolution of the Be-Be dimer that is capping one side of the distorted ring boron along with the IRC of the chiral Be4 B8 cluster. Panel (**b**) shows the evolution of distance between the two dimers that are capping the distorted ring boron along with the IRC of the chiral Be4 B8 cluster. In panel (**a**), the minimum Be-Be bond length is located at TS state with a value of 1.9416 Å, and the maximum value is 1.9862 Å that corresponds to one of the

putative global minima. The largest rate of decreasing/increasing bond length of Be-Be dimer is happening when the reaction start/end, before or after the maximum force point. (see video IRC).

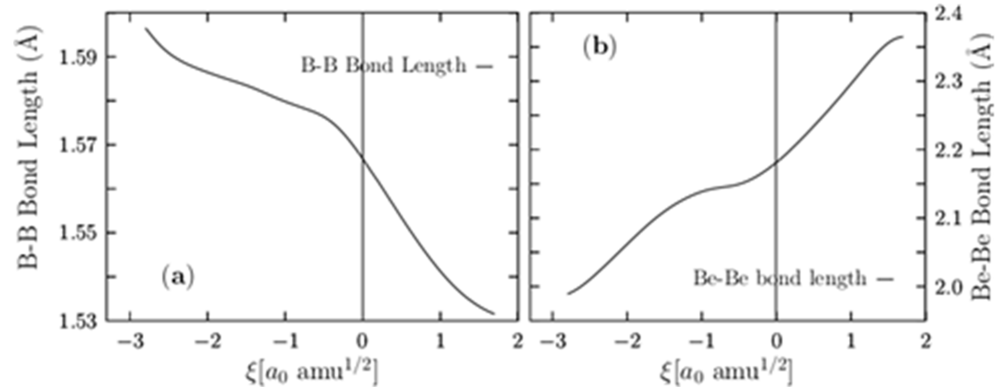

Figure S2. Panel (**a**) shows the bond length evolution of the B-B bond length along with the IRC of the chiral to achiral Be4B8 cluster. Panel (**b**) shows the evolution of distance between the two dimers that are capping the distorted ring boron along the IRC of the chiral to achiral Be_4B_8 cluster.

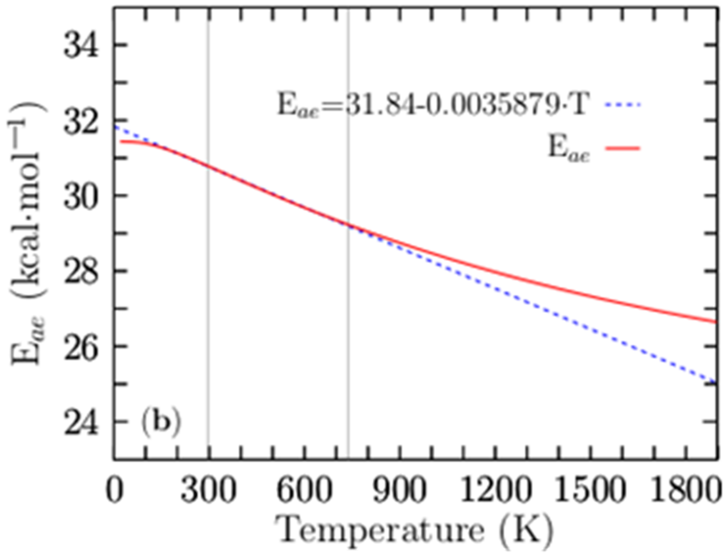

Figure S3. We show a straight line in the blue dashed line overlapping the energy barrier for enantiomers in the temperature range 200 to 740 K.

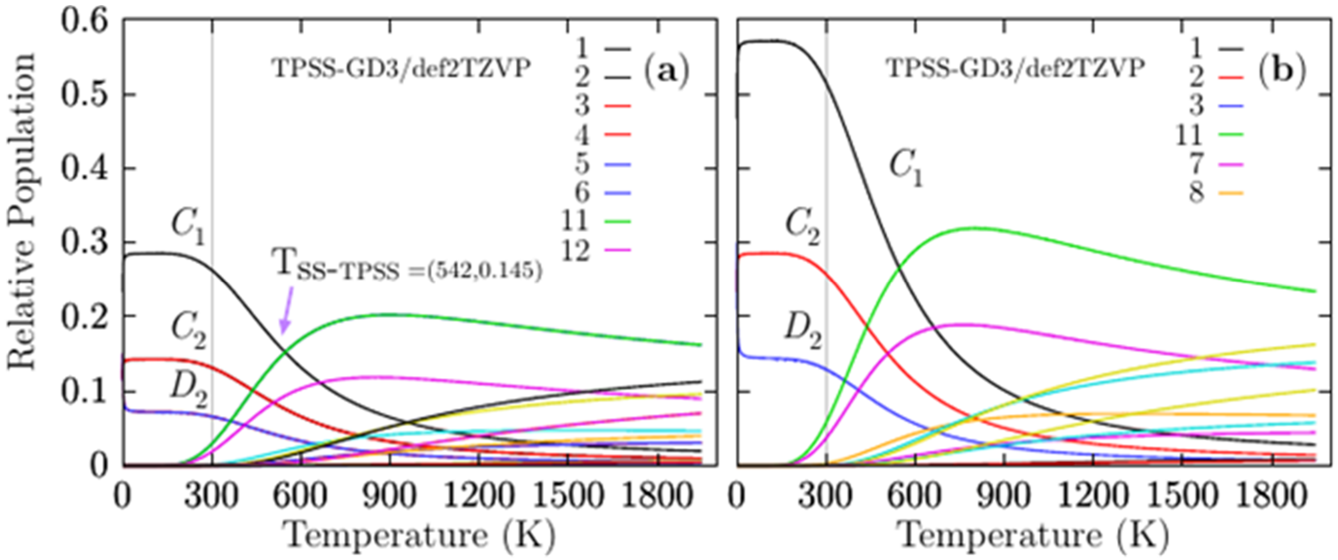

Figure S4. Probability occurrence of each isomer computed employing TSPP functional with the def2TZVP basis set, taking into account version three of Grimme's dispersion as it is implemented in Gaussian code. The relative energies between two isomers vary considerably with the functional use. This will affect the temperature-dependent Boltzmann factors computed for each isomer and, therefore, the relative population change, as shown in Figure. Employing TPSS functional, the TSS point is located at 542 K on a temperature scale compared with the T_{SS} point located at 739 K found employing PBE0 functional.

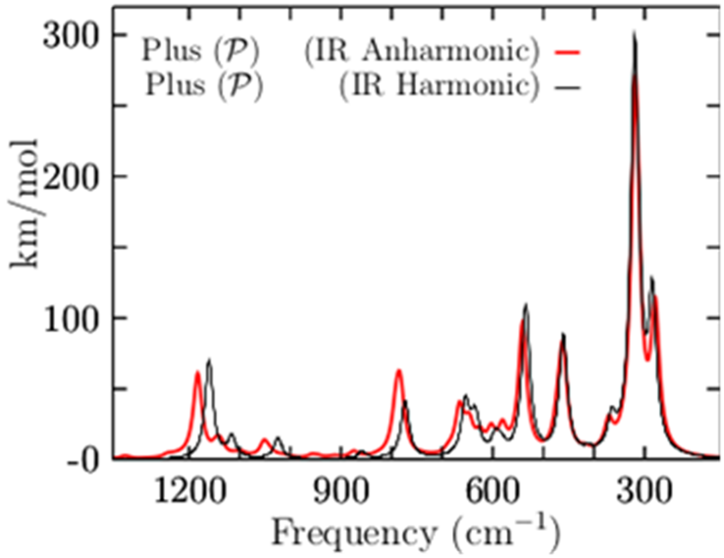

Figure S5. We show a comparison between IR Harmonic vs. IR Anharmonic spectra. IR-Harmonic spectrum was scaled by 0.96 to overlap the IR Anharmonic spectrum. The full width at half maximum (FWHM) employed is $20cm^{-1}$.

XYZ atomic coordinates

12

0.000000000 cluster_0001.out

Be -0.905655000000 -0.409167000000 1.143981000000

Be 0.906419000000 -0.409960000000 -1.144242000000

Be -0.905173000000 0.408402000000 -1.143806000000

Be 0.905131000000 0.410037000000 1.143131000000

B -0.682576000000 -1.497315000000 -0.425977000000

B -0.684257000000 1.498360000000 0.425282000000

B 0.684218000000 -1.498348000000 0.426351000000

B 0.682942000000 1.498649000000 -0.425686000000

B -2.072794000000 0.728212000000 0.251748000000

B 2.072272000000 -0.728536000000 0.252462000000

B 2.071532000000 0.729531000000 -0.251631000000

B -2.071915000000 -0.730003000000 -0.251800000000

12

0.000000000 cluster_0002.out

Be -0.905655000000 -0.409167000000 -1.143981000000

Be 0.906419000000 -0.409960000000 1.144242000000

Be -0.905173000000 0.408402000000 1.143806000000

Be 0.905131000000 0.410037000000 -1.143131000000

B -0.682576000000 -1.497315000000 0.425977000000

B -0.684257000000 1.498360000000 -0.425282000000

B 0.684218000000 -1.498348000000 -0.426351000000

B 0.682942000000 1.498649000000 0.425686000000

B -2.072794000000 0.728212000000 -0.251748000000

B 2.072272000000 -0.728536000000 -0.252462000000

B 2.071532000000 0.729531000000 0.251631000000

B -2.071915000000 -0.730003000000 0.251800000000

12

0.410385000 cluster_0003.out

Be 1.143790000000 0.906443000000 -0.409564000000

Be -1.143790000000 -0.906443000000 -0.409564000000

Be -1.143790000000 0.904746000000 0.409220000000

Be 1.143790000000 -0.904746000000 0.409220000000

B -0.426406000000 0.683245000000 -1.497832000000

B 0.425241000000 0.683751000000 1.498505000000

B 0.426406000000 -0.683245000000 -1.497832000000

B -0.425241000000 -0.683751000000 1.498505000000

B 0.250954000000 2.072252000000 0.728872000000

B 0.252867000000 -2.072004000000 -0.729270000000

B -0.250954000000 -2.072252000000 0.728872000000

B -0.252867000000 2.072004000000 -0.729270000000

12

0.410385000 cluster_0004.out

Be -1.143790000000 0.906443000000 -0.409564000000

Be 1.143790000000 -0.906443000000 -0.409564000000

Be 1.143790000000 0.904746000000 0.409220000000

Be -1.143790000000 -0.904746000000 0.409220000000

B 0.426406000000 0.683245000000 -1.497832000000

B -0.425241000000 0.683751000000 1.498505000000

B -0.426406000000 -0.683245000000 -1.497832000000

B 0.425241000000 -0.683751000000 1.498505000000

B -0.250954000000 2.072252000000 0.728872000000

B -0.252867000000 -2.072004000000 -0.729270000000

B 0.250954000000 -2.072252000000 0.728872000000

B 0.252867000000 2.072004000000 -0.729270000000

12

0.818260000 cluster_0005.out

Be 0.905773000000 0.409561000000 1.144321000000

Be -0.905773000000 0.409561000000 -1.144321000000

Be 0.905773000000 -0.409561000000 -1.144321000000

Be -0.905773000000 -0.409561000000 1.144321000000

B 0.683419000000 1.497675000000 -0.426202000000

B 0.683419000000 -1.497675000000 0.426202000000

B -0.683419000000 1.497675000000 0.426202000000

B -0.683419000000 -1.497675000000 -0.426202000000

B 2.072105000000 -0.728873000000 0.252168000000

B -2.072105000000 0.728873000000 0.252168000000

B -2.072105000000 -0.728873000000 -0.252168000000

B 2.072105000000 0.728873000000 -0.252168000000

12

0.818260000 cluster_0006.out

Be -0.905773000000 0.409561000000 1.144321000000

Be 0.905773000000 0.409561000000 -1.144321000000

Be -0.905773000000 -0.409561000000 -1.144321000000

Be 0.905773000000 -0.409561000000 1.144321000000

B -0.683419000000 1.497675000000 -0.426202000000

B -0.683419000000 -1.497675000000 0.426202000000

B 0.683419000000 1.497675000000 0.426202000000

B 0.683419000000 -1.497675000000 -0.426202000000

B -2.072105000000 -0.728873000000 0.252168000000

B 2.072105000000 0.728873000000 0.252168000000

B 2.072105000000 -0.728873000000 -0.252168000000

B -2.072105000000 0.728873000000 -0.252168000000

12

1.792140000 cluster_0007.out

Be -2.202732000000 0.773468000000 0.000000000000

Be 0.148526000000 -1.149908000000 1.103364000000

B -1.302324000000 0.094462000000 1.381693000000

B -1.281600000000 -0.848034000000 0.000000000000

B 1.508523000000 0.192109000000 1.453211000000

B 0.114703000000 0.434511000000 2.039453000000

Be 0.148526000000 -1.149908000000 -1.103364000000

Be 0.162086000000 0.773968000000 0.000000000000

B 1.508523000000 0.192109000000 -1.453211000000

B 0.114703000000 0.434511000000 -2.039453000000

B 2.034673000000 0.007775000000 0.000000000000

B -1.302324000000 0.094462000000 -1.381693000000

12

2.403325000 cluster_0008.out

Be 1.331477000000 -1.048647000000 1.048028000000

Be -0.549187000000 0.000009000000 1.135133000000

B -0.113798000000 -1.702898000000 -0.146321000000

B -1.616633000000 1.395580000000 0.016192000000

B -2.270990000000 0.000025000000 0.111955000000

B -1.616712000000 -1.395551000000 0.016052000000

Be 1.331425000000 1.047987000000 1.048446000000

Be -0.641371000000 -0.000023000000 -0.997149000000

B 1.148833000000 -0.895328000000 -0.779258000000

B 2.256135000000 -0.000009000000 -0.080882000000

B -0.113743000000 1.702968000000 -0.146229000000

B 1.149033000000 0.895752000000 -0.779075000000

12

4.459642500 cluster_0010.out

Be 0.928824000000 0.707602000000 1.175274000000

B 0.223228000000 1.870886000000 -0.340290000000

Be -0.837427000000 -0.477870000000 1.202727000000

B -1.798525000000 -1.100823000000 -0.326190000000

B 1.083266000000 -0.912714000000 -0.766825000000

B 1.427804000000 0.907097000000 -0.666957000000

B -1.112051000000 1.511182000000 0.331188000000

B -2.142394000000 0.326988000000 0.111709000000

Be -0.546764000000 0.194549000000 -0.980014000000

Be 1.062772000000 -1.359833000000 1.007188000000

B 2.161391000000 -0.215633000000 0.176572000000

B -0.328643000000 -1.638541000000 -0.443347000000

12

4.459642500 cluster_0009.out

Be 0.928824000000 0.707602000000 -1.175274000000

B 0.223228000000 1.870886000000 0.340290000000

Be -0.837427000000 -0.477870000000 -1.202727000000

B -1.798525000000 -1.100823000000 0.326190000000

B 1.083266000000 -0.912714000000 0.766825000000

B 1.427804000000 0.907097000000 0.666957000000

B -1.112051000000 1.511182000000 -0.331188000000

B -2.142394000000 0.326988000000 -0.111709000000

Be -0.546764000000 0.194549000000 0.980014000000

Be 1.062772000000 -1.359833000000 -1.007188000000

B 2.161391000000 -0.215633000000 -0.176572000000

B -0.328643000000 -1.638541000000 0.443347000000

12

4.702485000 cluster_0012.out

Be -0.774952000000 0.741777000000 -1.420369000000

B -0.508784000000 1.708828000000 0.741523000000

B -0.328288000000 0.685985000000 1.900401000000

B 0.328288000000 2.046944000000 -0.529636000000

B 1.093221000000 1.021365000000 -1.360156000000

Be 1.039020000000 0.502806000000 0.480205000000

Be -1.039020000000 -0.502806000000 0.480205000000

Be 0.774952000000 -0.741777000000 -1.420369000000

B 0.328288000000 -0.685985000000 1.900401000000

B -1.093221000000 -1.021365000000 -1.360156000000

B -0.328288000000 -2.046944000000 -0.529636000000

B 0.508784000000 -1.708828000000 0.741523000000

12

4.702485000 cluster_0011.out

Be 0.774952000000 0.741777000000 -1.420369000000

B 0.508784000000 1.708828000000 0.741523000000

B 0.328288000000 0.685985000000 1.900401000000

B -0.328288000000 2.046944000000 -0.529636000000

B -1.093221000000 1.021365000000 -1.360156000000

Be -1.039020000000 0.502806000000 0.480205000000

Be 1.039020000000 -0.502806000000 0.480205000000

Be -0.774952000000 -0.741777000000 -1.420369000000

B -0.328288000000 -0.685985000000 1.900401000000

B 1.093221000000 -1.021365000000 -1.360156000000

B 0.328288000000 -2.046944000000 -0.529636000000

B -0.508784000000 -1.708828000000 0.741523000000

12

4.722565000 cluster_0013.out

B 0.059240000000 1.766146000000 -0.000260000000

B -1.119754000000 -0.905597000000 -0.828650000000

B -2.254108000000 -0.142411000000 0.000626000000

B 2.274332000000 0.051830000000 -0.000386000000

Be 0.599786000000 0.017984000000 1.057705000000

B -1.118571000000 -0.905929000000 0.828784000000

Be 0.597170000000 0.018269000000 -1.055682000000

Be -1.371969000000 1.042516000000 0.990041000000

B 1.680118000000 -1.361677000000 -0.000979000000

Be -1.374317000000 1.044646000000 -0.989162000000

B 0.155429000000 -1.638937000000 -0.000940000000

B 1.562778000000 1.437845000000 -0.000518000000

12

6.000782500 cluster_0014.out

Be -0.000831000000 -1.504197000000 1.163549000000

B -0.000992000000 -1.850496000000 -0.686190000000

B -1.377473000000 -1.290750000000 -0.179707000000

B 1.376071000000 -1.292182000000 -0.179756000000

B -2.030013000000 0.135795000000 -0.480628000000

Be -1.153264000000 0.122468000000 1.201188000000

Be 0.000087000000 0.176750000000 -0.768636000000

Be 1.153201000000 0.121392000000 1.201216000000

B -1.456574000000 1.503183000000 -0.131881000000

B 0.001126000000 2.025999000000 0.032664000000

B 1.458280000000 1.501672000000 -0.131828000000

B 2.030217000000 0.133649000000 -0.480527000000

12

7.358065000 cluster_0015.out

Be 1.152144000000 -0.164443000000 1.060121000000

Be -0.001452000000 -1.817972000000 1.269345000000

B 0.861637000000 -1.576819000000 -0.283874000000

B 1.862077000000 0.984996000000 -0.332089000000

B 1.866049000000 -0.493594000000 -0.773120000000

Be 0.000289000000 0.308751000000 -0.830512000000

Be -1.152685000000 -0.162813000000 1.060032000000

B 0.778441000000 1.816072000000 0.365500000000

B -0.775435000000 1.817106000000 0.365598000000

B -0.864266000000 -1.575670000000 -0.283814000000

B -1.860370000000 0.987827000000 -0.332146000000

B -1.866772000000 -0.490737000000 -0.773244000000

12

7.407637500 cluster_0017.out

Be 0.878149000000 0.367822000000 1.209776000000

B 0.768782000000 -1.526024000000 0.379145000000

B 0.779324000000 -0.196292000000 -0.770992000000

B 2.088103000000 -0.700264000000 0.173432000000

Be -0.851058000000 1.630913000000 0.687801000000

Be -0.915176000000 0.610588000000 -1.082292000000

B 0.658956000000 1.516682000000 -0.436342000000

B -2.021939000000 -0.785016000000 -0.403273000000

Be -0.990017000000 -0.462802000000 1.193256000000

B -2.258953000000 0.573447000000 0.229924000000

B -0.557266000000 -1.404806000000 -0.500510000000

B 2.045475000000 0.805057000000 -0.278216000000

12

7.407637500 cluster_0016.out

Be -0.878149000000 0.367822000000 1.209776000000

B -0.768782000000 -1.526024000000 0.379145000000

B -0.779324000000 -0.196292000000 -0.770992000000

B -2.088103000000 -0.700263000000 0.173432000000

Be 0.851059000000 1.630913000000 0.687801000000

Be 0.915176000000 0.610588000000 -1.082292000000

B -0.658955000000 1.516682000000 -0.436342000000

B 2.021939000000 -0.785017000000 -0.403273000000

Be 0.990017000000 -0.462802000000 1.193256000000

B 2.258953000000 0.573446000000 0.229924000000

B 0.557266000000 -1.404806000000 -0.500510000000

B -2.045475000000 0.805058000000 -0.278216000000

12

8.007527500 cluster_0019.out

Be 0.937839000000 -0.461077000000 -1.149365000000

Be 0.660153000000 1.626387000000 -0.715075000000

B 2.169455000000 0.665689000000 -0.319997000000

B -0.649467000000 -1.510307000000 -0.364437000000

B -0.777048000000 0.223600000000 -0.820156000000

B 0.703991000000 -1.384969000000 0.537302000000

B 2.115532000000 -0.694855000000 0.356286000000

Be -0.878152000000 -0.334892000000 1.151016000000

Be 0.917148000000 0.717311000000 1.086115000000

B -0.757556000000 1.512408000000 0.457377000000

B -2.082007000000 0.732810000000 0.148087000000

B -2.032491000000 -0.782559000000 -0.292615000000

12

8.007527500 cluster_0018.out

Be -0.937839000000 -0.461077000000 -1.149365000000

Be -0.660153000000 1.626387000000 -0.715075000000

B -2.169455000000 0.665689000000 -0.319997000000

B 0.649467000000 -1.510307000000 -0.364437000000

B 0.777048000000 0.223600000000 -0.820156000000

B -0.703991000000 -1.384969000000 0.537302000000

B -2.115532000000 -0.694855000000 0.356286000000

Be 0.878152000000 -0.334892000000 1.151016000000

Be -0.917148000000 0.717311000000 1.086115000000

B 0.757556000000 1.512408000000 0.457377000000

B 2.082007000000 0.732810000000 0.148087000000

B 2.032491000000 -0.782559000000 -0.292615000000

12

9.165265000 cluster_0021.out

B -0.682366000000 -1.634716000000 -0.241046000000

B -1.832843000000 -0.709594000000 -0.747680000000

Be 0.006199000000 0.247296000000 -0.796127000000

B 0.196319000000 -1.444815000000 1.087095000000

Be 1.316793000000 -0.029415000000 1.029456000000

B 1.059454000000 -1.482850000000 -0.250268000000

B -1.009864000000 1.722706000000 0.278839000000

B 1.789309000000 1.217850000000 -0.325538000000

B -2.009766000000 0.773636000000 -0.389353000000

Be 2.234425000000 -0.352985000000 -0.953170000000

Be -1.132850000000 -0.221603000000 1.053560000000

B 0.550104000000 1.843149000000 0.320976000000

12

9.165265000 cluster_0020.out

B -0.682364000000 -1.634717000000 0.241046000000

B -1.832842000000 -0.709596000000 0.747680000000

Be 0.006199000000 0.247296000000 0.796127000000

B 0.196320000000 -1.444815000000 -1.087095000000

Be 1.316793000000 -0.029414000000 -1.029456000000

B 1.059455000000 -1.482849000000 0.250268000000

B -1.009866000000 1.722705000000 -0.278839000000

B 1.789308000000 1.217852000000 0.325538000000

B -2.009767000000 0.773634000000 0.389353000000

Be 2.234425000000 -0.352983000000 0.953170000000

Be -1.132850000000 -0.221604000000 -1.053560000000

B 0.550102000000 1.843150000000 -0.320976000000

12

9.201032500 cluster_0022.out

B 0.420735000000 -1.010176000000 1.305320000000

B 0.420735000000 1.871253000000 0.783131000000

Be -1.371267000000 -0.601739000000 1.160674000000

Be 1.080577000000 0.317060000000 0.000000000000

B 0.420735000000 -1.010176000000 -1.305320000000

B -0.089080000000 0.629982000000 -1.611027000000

Be -1.124461000000 1.125902000000 0.000000000000

B -0.454293000000 -1.659055000000 0.000000000000

B 1.178649000000 -1.514652000000 0.000000000000

Be -1.371267000000 -0.601739000000 -1.160674000000

B -0.089080000000 0.629982000000 1.611027000000

B 0.420735000000 1.871253000000 -0.783131000000
